# Freezing Protocol Optimization for Iberian Red Deer (*Cervus elaphus hispanicus*) Epididymal Sperm under Field Conditions

**DOI:** 10.3390/ani12070869

**Published:** 2022-03-30

**Authors:** Daniela Alejandra Medina-Chávez, Ana Josefa Soler, Alicia Martín-Maestro, Silvia Villaverde, Irene Sánchez-Ajofrín, Patricia Peris-Frau, Enrique del Olmo, Alfonso Bisbal, Olga García-Álvarez, María del Rocío Fernández-Santos, José Julián Garde

**Affiliations:** 1 SaBio IREC (CSIC-UCLM-JCCM), ETSIAM, Campus Universitario s/n, 02071 Albacete, Spain; daniela.medina@uclm.es (D.A.M.-C.); alicia.martinmaestro@uclm.es (A.M.-M.); silvia.villaverde@alu.uclm.es (S.V.); irene.ssanchez@uclm.es (I.S.-A.); patricia.perisfrau@gmail.com (P.P.-F.); enrique.delolmo@uclm.es (E.d.O.); alfonso.bisbal@uclm.es (A.B.); olga.garcia@uclm.es (O.G.-Á.); mrocio.fernandez@uclm.es (M.d.R.F.-S.); julian.garde@uclm.es (J.J.G.); 2 Instituto Regional de Investigación Científica Aplicada (IRICA), Universidad de Castilla-La Mancha (UCLM), 13071 Ciudad Real, Spain; 3 Department Animal Reproduction (INIA-CSIC), Avenida Puerta de Hierro Km 5.9, 28040 Madrid, Spain

**Keywords:** cryopreservation, epididymal sperm, Iberian red deer, field conditions

## Abstract

**Simple Summary:**

The germplasm banks of wild species, such as Iberian red deer, are not widespread, mainly due to the difficulties of collecting and cryopreserving reproductive cells. Optimal freezing protocols under field conditions could be a breakthrough for these species. In this study, epididymal sperm was evaluated using two methods of sperm storage during refrigeration (tube and straw); four equilibration periods (0, 30, 60, and 120 min); and four methods of freezing (cryopreservation in liquid nitrogen vapors in a tank (control) or box, freezing in dry ice, or freezing over a metallic plate). The results showed that samples stored in straws during refrigeration produced less apoptotic spermatozoa and more viable spermatozoa withactive mitochondria. A long equilibration period (120 min) yielded a higher percentage of acrosomal integrity. Moreover, there was no difference in sperm quality between freezing in liquid nitrogen vapors in a tank or box. However, a worse quality was obtained when the samples were cryopreserved in dry ice or over a metallic plate compared to the control.

**Abstract:**

Creating germplasm banks of wild species, such as the Iberian red Deer (*Cervus elaphus hispanicus*) can be challenging. One of the main difficulties is the obtention and cryopreservation of good-quality reproductive cells when the spermatozoa are obtained from epididymides after death. To avoid a loss of seminal quality during transport, developing alternative methods for cooling and freezing sperm samples under field conditions is necessary. The objective of this study was to evaluate the effects of different durations of equilibrium and different techniques of cooling and freezing on Iberian red deer epididymal sperm quality after thawing to optimize the processing conditions in this species. Three experiments were carried out: (I) evaluation of refrigeration in straws or tubes of 15 mL; (II) study of equilibration period (0, 30, 60, or 120 min); and (III) comparison of four freezing techniques (liquid nitrogen vapor in a tank (C), liquid nitrogen vapor in a polystyrene box (B), dry ice (DY), and placing straws on a solid metallic plate floating on the surface of liquid nitrogen (MP)). For all experiments, sperm motility and kinematic parameters, acrosomal integrity, sperm viability, mitochondrial membrane potential, and DNA integrity were evaluated after thawing. All statistical analyses were performed by GLM-ANOVA analysis. Samples refrigerated in straws showed higher values (*p* ≤ 0.05) for mitochondrial activity and lower values (*p* ≤ 0.05) for apoptotic cells. Moreover, the acrosome integrity showed significant differences (*p* ≤ 0.05) between 0 and 120 min, but not between 30 and 60 min, of equilibration. Finally, no significant differences were found between freezing in liquid nitrogen vapors in a tank or in a box, although there was a low quality after thawing when the samples were cryopreserved in dry ice or by placing straws on a solid metallic plate floating on the surface of liquid nitrogen. In conclusion, under field conditions, it would be possible to refrigerate the sperm samples by storing them in straws with a 120 min equilibration period and freezing them in liquid nitrogen vapors in a tank or box.

## 1. Introduction

In the last few decades, progress has been made in establishing Genome Resource Banks (GRBs), which ensure long-term genetic preservation and variability and improve the reproductive efficiency of wild species and domestic animals [1,2,3,4,5,6,7,8,9]. This progress is mainly due to the development of new techniques of sperm cryopreservation, using specifical freezing media or freezing rates, which altogether have improved sperm cryosurvival [10,11]. However, sperm cryopreservation is a complex process that involves many factors, both cellular (e.g., shape, size, membrane lipid composition, sperm source) and dependent on the freezing protocol (e.g., cooling and freezing rates or use of cryoprotectants), that may entail some risks of sperm injury related to osmotic, biochemical, and physicochemical intracellular changes, which hinder the storage and preservation of sperm reproductive potential [9,11,12,13,14]. Some studies have described the influence of several factors, from the type of species [11] to methodological aspects, such as the use of permeable and nonpermeable cryoprotective agents [6,15], cooling and thawing techniques [16,17,18,19], and different methods of semen collection [19,20], on post-thaw sperm viability. Thus, these studies highlight that, to ensure the survival and viability of the spermatozoa, all factors involved in semen cryopreservation must be considered.

Lately, there has been significant interest in using artificial reproductive technologies (ARTs) for the handling of Iberian red deer (*Cervus elaphus hispanicus*; Hilzheimer, 1909) populations, not only because of their livestock and recreational hunting interest [6], but also because they could be used as a model for other related endangered subspecies. Notably, in wild deer populations within fenced hunting estates, inbreeding has led to genetic isolation and has resulted in detrimental effects on some components of female fitness and male reproductive ability [21]. In this situation, the cryopreservation of Iberian red deer epididymal spermatozoa has offered the possibility of progressing in establishing genetic resource banks for this species, since they can be obtained from certain types of hunting [6,7,22,23,24,25,26,27,28]. Some studies have demonstrated that it is possible to obtain viable sperm from the epididymis 24 h after death if the testis is stored at room temperature [29], or up to 4 days if held at 5 °C [26]. The viability of epididymal spermatozoa has been surprisingly high even after freezing and thawing [27]. However, although progress has been made in the cryopreservation of deer epididymis semen, improvements are still needed to maximize its quality after freeze–thaw protocols. This is because most of the protocols used in the cryopreservation of the epididymis sperm have been adapted from those used in ejaculate sperm, despite the physiological differences that these present [6]. 

Therefore, all semen cryopreservation factors, parameters, and phases are required to assure sperm viability and to develop specific protocols in Iberian red deer epididymal spermatozoa. The transport containers, diluents, storage techniques during refrigeration, cryoprotectant agent (CPA), cooling rate, equilibration period, and freezing and thawing protocols are the key to success in terms of sperm survival.

It has been seen that packing techniques during freezing could affect post-thaw sperm quality in different species [30,31,32], and that the surface-to-volume ratio determined by the method used seems to be decisive [33]. However, there are no studies that have evaluated the effects of different storage methods during the refrigeration of sperm samples on their quality.

On the other hand, the effects of the equilibration time have been studied in other species [34,35], with a variety of results depending on the type and concentration of the cryoprotectant [36]. The equilibration period encourages sperm membrane stability, mainly in the acrosomal membrane, due to the adaptation of membrane lipids to cooler temperatures [35,37]. Thus, facilitating the movement of the penetrating cryoprotectants through the membrane and allowing water outlet to the outside of the cell minimizes the ice formation during the freeze–thaw process and diminishes possible damage [38]. Several studies have been conducted on semen from different species, including sheep [39], goats [40], and cattle [41], to identify the optimum equilibrium period. Although in Iberian red deer sperm the usual equilibration period used in freeze–thaw protocols is 120 min [6], there is a lack of studies about the effects of different equilibration periods on the post-thaw epididymal sperm quality in this species.

Apart from this, the effects of freezing methods on sperm quality have been widely studied in various species, with different results among them [42]. Some studies on ungulates have shown the effects of storage temperature on epididymal and ejaculated semen [43,44] and the effects of the cooling rate on the freezability of Iberian red deer sperm [6]. 

Most Iberian red deer samples are obtained from locations far from the laboratory and may decrease the sperm quality before processing. A possible solution could be to perform the freezing process in the field. However, sperm freezing requires large apparatus that cannot be transported to the sample collection site, so the use of protocols adapted to field conditions would be a possible solution to avoid decreasing the quality of the samples.

Bearing all this in mind, the overall objective of this work was to develop a specific protocol for the cryopreservation of Iberian red deer epididymal spermatozoa that improves the outcome and can be used under field conditions. In this regard, three different experiments were developed: (1) to determine the effects of two storage techniques during the cooling phase, (2) to explore the action of four equilibration periods, and (3) to evaluate possible alternatives to the conventional method of cryopreservation in liquid nitrogen vapors.

## 2. Materials and Methods

### 2.1. Chemicals and Solutions

Unless otherwise indicated, all the reagents were purchased from Sigma-Aldrich (Madrid, Spain). Fluorescent stocks were prepared in DMSO, according to the specifications of the fluorochrome, and placed in the dark at −20 °C until needed. The freezing extender was split into two fractions: fraction A and fraction B. Fraction A contained Tris–citrate–fructose and clarified egg yolk (EY 20%), and fraction B had the same composition plus addition of permeant cryoprotector (glycerol 12%) [6]. Bovine gamete medium (BGM-3) was composed of: 87 mmol/L NaCl; 3.1 mmol/L KCl; 2 mmol/L CaCl2; 0.4 mmol/L MgCl2; 0.3 mmol/L NaH2PO4; 40 mmol/L HEPES; 21.6 mmol/L sodium lactate; 1 mmol/L sodium pyruvate; 50 μg/mL kanamycin; 10 μg/mL phenol red; and 6 mg/mL BSA (bovine serum albumin) (pH 7.5) [45].

### 2.2. Testes and Sperm Collection

Testes were collected from 25 Iberian red deer for each experiment. All the animals were adults and were hunted legally during the rutting season in Castilla-La Mancha (Spain) within the harvest plan of the game reserve according to the Spanish Harvest Regulation, Law 2/93 of Castilla-La Mancha, which conforms to European Union regulations. The testes were collected 6 h after slaughter and transported to the laboratory in plastic bags at room temperature (approximately 15 °C). Testes were then removed from the scrotal sac, and caudal epididymides were separated and transferred into a petri dish.

### 2.3. Sperm Processing and Fresh Quality Evaluation

Sperm were collected from distal epididymis according to the method described by Soler et al. [46] and diluted in an exact volume (0.5 mL) of freezing medium fraction A (Tris–citrate–fructose and clarified egg yolk 20%). Subsequently, sperm concentration was assisted with a Neubauer chamber (Marienfeld, Lauda-Königshofen, Germany). In addition, sperm motility was assessed for each sample as described below. Only ejaculates with sperm motility higher than 60% were cryopreserved.

### 2.4. Cryopreservation of Epidydimal Spermatozoa

**Figure 1:**
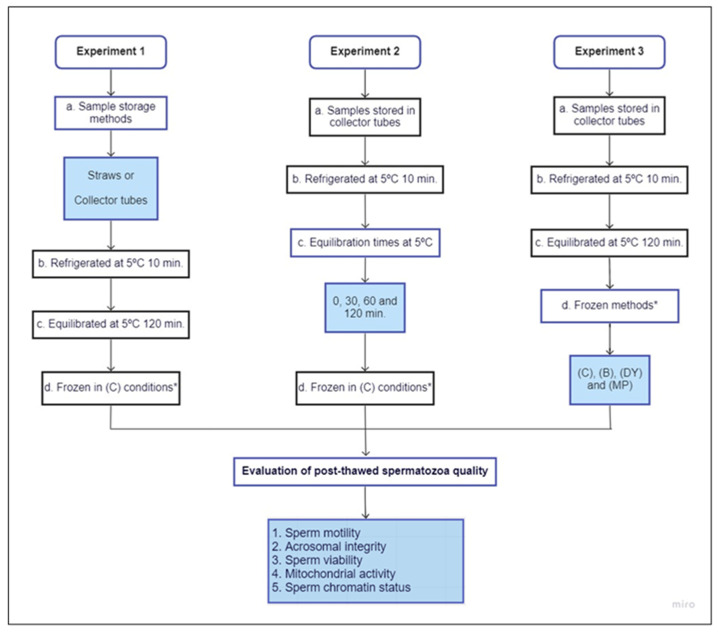
Experimental design: (C) control—samples frozen in liquid nitrogen vapors in tank; (B) box—samples frozen in liquid nitrogen vapor in polystyrene box; (DY) dry ice—samples frozen in dry ice inside a polystyrene box; (MP) metallic plate—samples frozen in a solid metallic plate floating on the surface of liquid nitrogen. * Note that the samples refrigerated in 15 mL collector tubes, before being frozen, were also loaded in 0.25 mL plastic straws.

Briefly, sperm was again diluted in a two-step procedure: first, semen was diluted to 400 × 10^6^ spermatozoa/mL with fraction A, and then fraction B was added until achieving a final concentration of spermatozoa (200 × 10^6^ spermatozoa/mL) and glycerol (6%). Both steps took place at room temperature. After dilution, the study was subdivided into three experiments with the following experimental design (Figure 1). Experiment 1 evaluated different storage techniques during sample refrigeration. Experiment 2 examined the effect of different equilibration times, and Experiment 3 determined the effect of several freezing techniques on post-thaw sperm quality. 

#### 2.4.1. Experiment 1: Evaluation of the Spermatozoa Quality in Iberian Red Deer Epididymal Samples Refrigerated in Collector Tubes of 15 mL or 0.25 mL straws

To determine the effects of two different storage techniques, two aliquots of each deer were treated and diluted as described above. Once diluted, one aliquot was refrigerated in a 15 mL collector tube immersed in water at 5 °C, and the other was refrigerated in a straw immersed in water at 5 °C. Both samples were refrigerated for 10 min until the temperature of 5 °C was reached. Then, samples were kept at this temperature for 120 min and frozen in liquid nitrogen vapor in a tank. Note that the samples refrigerated in 15 mL collector tubes, before being frozen, were also loaded in 0.25 mL plastic straws.

#### 2.4.2. Experiment 2: Effect of Different Equilibrium Times on Post-Thaw Sperm Quality of Iberian Red Deer Spermatozoa

To evaluate several equilibration times and their effects on post-thaw spermatozoa survival, four aliquots of every stag were treated and diluted as described above. After dilution, samples were refrigerated at 5 °C for 10 min in a tube and maintained at this temperature for 0, 30, 60, or 120 min. Then, they were loaded into 0.25 mL plastic straws and frozen in liquid nitrogen vapor in a tank.

#### 2.4.3. Experiment 3: Effect of Freezing Techniques on Post-Thaw Sperm Quality of Iberian Red Deer Epididymal Spermatozoa

To search for the most suitable method under field conditions apart from a liquid nitrogen tank, three alternative freezing techniques were evaluated. In this experiment, four aliquots of every stag were used, and they were diluted as described above. Once diluted, samples were cooled to 5 °C for 10 min in a tube and held for 120 min at this temperature to equilibrate them. Following the equilibration period, aliquots were frozen with different methods. Four groups were conducted within this experiment: control (C), box (B), dry ice (DY), and metallic plate (MP). The C group was frozen by the standard methodology (liquid nitrogen vapor in a tank) [6]. The B group was frozen by placing the straws in a polystyrene box with liquid nitrogen, first at 4 centimeters above the liquid nitrogen for 10 min, and then dipped in the liquid nitrogen. The DY group was placed into a polystyrene box with dry ice inside it and maintained for 10 min directly on the dry ice; once this period had passed, straws were submerged into the liquid nitrogen. Finally, the MP group consisted of placing straws on a solid metallic plate floating on the surface of liquid nitrogen for 10 min; after this period, straws were submerged into the liquid nitrogen.

### 2.5. Thawing and Evaluation of Post-Thaw Spermatozoa Quality

The thawing procedure was accomplished by placing the straws in a 37 °C water bath for 20 sec. Sperm motility, acrosome status, and plasma membrane integrity were assessed for each sample to determine sperm quality in vitro.

#### 2.5.1. Sperm Motility Assays

All samples were evaluated for sperm motility by a computer-assisted sperm analyzer (CASA). A prewarmed (37 °C) Makler counting chamber (10 µm depth; Sefi Medical Instruments, Haifa, Israel) was loaded with 5 µL of the sample. The CASA system consisted of a trinocular optical phase-contrast microscope (Nikon Eclipse 80i, Nikon Instruments Inc, Tokyo, Japan) and a Basler A302fs digital camera (Basler Vision Technologies, Ahrensburg, Germany). The camera was connected to a computer by an IEEE 1394 interface. Images were captured and analyzed using the Sperm Class Analyzer (SCA 2002) software (Microptic S.L., Barcelona, Spain) adjusted to ram spermatozoa. The sample was examined with a 10× objective lens (negative phase contrast) in a microscope with a heated plate, and five areas were recorded. The following parameters were assessed: percentage of motile spermatozoa (SM); curvilinear velocity (VCL, µm s^−1^); straight-line velocity (VSL, µm s^−1^); average path velocity (VAP, µm s^−1^); linearity index (LIN, %); and amplitude of lateral head displacement (ALH, µm).

#### 2.5.2. Acrosomal Integrity Percentage

Acrosomal integrity was assessed by phase-contrast microscopy (Nikon Eclipse 80i, Nikon Instruments Inc, Tokyo, Japan) with a 400× objective lens. For this purpose, 5 µL of the diluted semen was fixed in 2% glutaraldehyde in 0.165 M cacodylate/HCl buffer at pH 7.3 (1:20 dilution). The percentage of spermatozoa with intact acrosomes (%NAR) was calculated by counting those with an intact apical rim. At least 100 cells of each sample were evaluated. 

#### 2.5.3. Assessment of Sperm Viability and Mitochondrial Activity 

Analyses of sperm viability, as well as mitochondrial activity, were performed using YO-PRO-1/propidium iodide (IP), and Mitotracker Deep Red (MT)/YO-PRO-1, respectively [47]. Briefly, the samples were diluted to a concentration of 10^6^ spermatozoa/mL in BGM-3 solution and stained using the fluorophores. Sperm viability was assessed with 0.1 μM YO-PRO-1 (Invitrogen, Barcelona, Spain) and 10 μM PI, whereas mitochondrial membrane potential was assessed with 0.1 μM YO-PRO-1 and 0.1 μM MT. The tubes were left to rest for 20 min in the dark and then analyzed by flow cytometry.

The samples were run through a flow cytometer (Cytomics FC500; Becton Dickinson, San José, California) furnished with a 488 nm Argon-Ion laser (excitation for YO-PRO-1 and PI), and a 635 nm He–Ne laser (excitation for MT). The FSC (forward-scattered light) and SSC (side-scattered light) signals were used to gate out debris (non-sperm events). Fluorescence from YO-PRO-1, PI, and MT was read using a 525/25BP, 615DSP, and 675/40BP filter, respectively. All the parameters were read using logarithmic amplification. Ten thousand spermatozoa of each sample were recorded. Flow cytometer data were analyzed by WEASEL v2.6 (WEHI; Melbourne, Victoria, Australia) software using the following guidelines: the YO-PRO-1-/PI- and YO-PRO-1+/PI- sperm subpopulations were considered viable spermatozoa with an intact membrane and apoptotic spermatozoa, respectively, and the YO-PRO-1-/MT+ sperm subpopulation was considered as live, with active mitochondria.

#### 2.5.4. Sperm Chromatin Assessment

Samples were diluted in TNE buffer (0.01 M Tris–HCl, 0.15 M NaCl, 1 mM EDTA, pH 7.4) to a final sperm concentration of 2 × 10^6^ spermatozoa/mL and immediately frozen in liquid nitrogen. Samples were stored at −80 °C until use. Chromatin stability was assessed following the SCSA^®^ (Sperm Chromatin Structure Assay; SCSA diagnostics, Brookings, SD, USA). This test measures the percentage of sperm with fragmented DNA and the degree of DNA damage [48]. For analysis by flow cytometry, samples were thawed in a 37 °C water bath, and 200 µL of the sperm sample was submitted to a DNA denaturation step by adding 0.4 mL of an acid–detergent solution (0.17% Triton X-100, 0.15 M NaCl, 0.08 N HCl, pH 1.4). After 30 seconds, samples were mixed with 1.2 mL of acridine orange (AO) solution (0.1 M citric acid, 0.2 M Na2HPO4, 1 mM EDTA, 0.15 M NaCl, 6 µg/mL AO, pH 6.0) and analyzed by flow cytometry after two and a half minutes. AO is a metachromatic fluorochrome that shifts from green (dsDNA, double-strand) to red (ssDNA, single-strand) depending on the degree of DNA denaturation. Samples were run through Cytomics FC500, as described above, using the 488 nm laser and 530/28BP filter for green fluorescence and 620SP filter for red fluorescence. The DNA fragmentation index (% DFItotal) was measured for every sample to show the amount of red emission produced by a sample regarding total fluorescence emitted.

### 2.6. Statistical Analysis

All statistical analyses were performed using SPSS for Windows, version 22.0 (SYSTAT Software Inc., Evanston, IL, USA). A generalized linear means (GLM-ANOVA) model that included the method of refrigeration (tube or straw), equilibration time (0, 30, 60, or 120 min), and freezing methods (in liquid nitrogen vapors in a tank or box, in dry ice, or on a metallic plate) as fixed factors and the sperm quality parameters as an independent variable was constructed to study the significant differences in post-thaw sperm quality parameters. Comparison of means was performed using the Bonferroni test. A *p*-value of ≤ 0.05 was considered statistically significant.

## 3. Results

**Figure 2:**
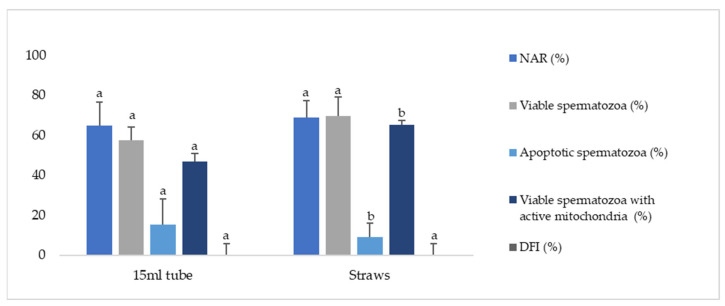
Effects of storage methods during refrigeration of Iberian red deer epididymal sperm samples on spermatozoa quality. Sperm parameters were assessed for two different storage techniques (tubes of 15 mL or 0.25 mL straws) during refrigeration. Data represented as mean ± SEM. NAR—acrosome integrity (%); viable (nonapoptotic) spermatozoa (%); apoptotic spermatozoa (%); viable spermatozoa with active mitochondria (%); DFI—DNA fragmentation index (%). Different letters indicate significant differences between treatments (*p* ≤ 0.05).

The first experiment was performed to determine the effects of the storage methods during refrigeration on sperm quality. We studied two different methods of storage: in collector tubes or straws. As reported, no significant differences were found in NAR and viability (nonapoptotic) when samples were refrigerated in straws instead of 15 mL collector tubes (Figure 2). Moreover, the DNA integrity was similar between the two forms of refrigeration. Nonetheless, apoptotic spermatozoa and viable spermatozoa with active mitochondria showed significant differences (*p* ≤ 0.05) between both treatments.

Regarding the kinematic sperm parameters, there were no differences for VCL, VSL, VAP, LIN, or ALH when samples were refrigerated in a tube or straw (Table 1). 

**Figure 3:**
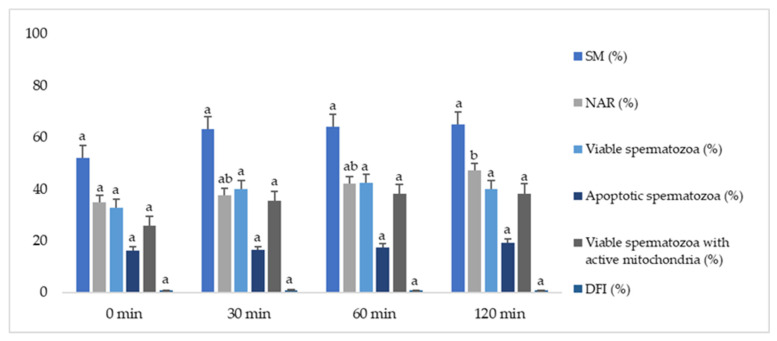
Effect of different equilibration times on the quality of thawed Iberian red deer epididymal spermatozoa. Sperm parameters were assessed for different equilibration times (0, 30, 60, and 120 minutes). Data represented as mean ± SEM. SM—sperm motility index (%); NAR—acrosome integrity (%); viable (nonapoptotic) spermatozoa (%); apoptotic spermatozoa (%); viable spermatozoa with active mitochondria (%); DFI—DNA fragmentation index (%). Different letters indicate significant differences between equilibration times (*p* ≤ 0.05).

In the second experiment, the same sperm parameters were studied according to different equilibration periods at 5 °C (0, 30, 60, and 120 min). No significant differences were found in SM, viability (nonapoptotic), apoptotic spermatozoa, viable spermatozoa with active mitochondria, and DNA integrity for 0, 30, 60, and 120 min (Figure 3). Nevertheless, NAR showed significant differences (*p* ≤ 0.05) between 0 and 120 min, with higher values for the longest refrigeration time. Moreover, 120 min of refrigeration yielded values of VSL and VAP higher in relation to 30 or 60 min of refrigeration or bypassing this process (Table 2). 

**Figure 4:**
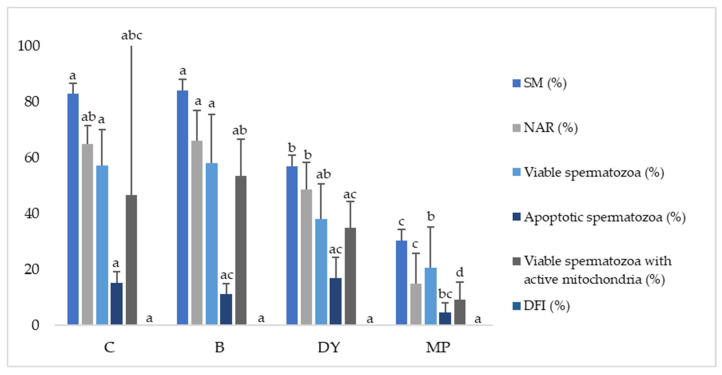
Effect of freezing techniques on post-thaw sperm quality in Iberian red deer epididymal spermatozoa. C (control—liquid nitrogen vapor in a tank), B (box—polystyrene box with liquid nitrogen inside), DY (dry ice—polystyrene box with dry ice inside), and MP (metallic plate—solid metallic plate floating on the surface of liquid nitrogen). Data represented as mean ± SEM. SM—sperm motility (%); NAR—acrosome integrity (%); viable (nonapoptotic) spermatozoa (%); apoptotic spermatozoa (%); viable spermatozoa with active mitochondria (%); DFI—DNA fragmentation index (%). Different letters indicate significant differences between freezing methods (*p* ≤ 0.05).

Finally, different freezing techniques were assessed to evaluate their effect on post-thaw sperm quality. No significant differences were found in SM and NAR for C and B (Figure 4). Nevertheless, SM and NAR showed significant differences (*p* ≤ 0.05) between these treatments and the remaining two groups (DY and MP), with the lowest values for MP. Concerning viability, apoptotic spermatozoa, and viable spermatozoa with active mitochondria, no significant differences were found between C, B, and DY, although these treatments were different (*p* ≤ 0.05) to MP, with the lowest values for this freezing method (Figure 4). However, the DNA integrity was not affected by the freezing procedure, with similar values between treatments. 

In the same way as for the other sperm parameters, VCL, VSL, VAP, and ALH showed lower (*p* ≤ 0.05) values for the MP freezing method compared to C, B, and DY (Table 3). 

## 4. Discussion

Given that a suitable freezing protocol for Iberian red deer epididymal spermatozoa under field conditions has not yet been published, this study focused for the first time on evaluating the effects of different storage methods during refrigeration, different equilibration times, and different freezing techniques on the quality of epididymal spermatozoa after thawing. All this was carried out with the aim of improving and simplifying the freezing conditions of epididymal spermatozoa in this species under field conditions.

In this way, Experiment 1 showed that refrigeration in straws seems to be associated with higher levels of viable spermatozoa with active mitochondria and a significantly lower percentage of apoptotic cells in post-thaw samples. These findings align with previous studies in other species, in which a lower volume of the samples packed before refrigeration improved the motility parameters and fertility [31]. A lower sample volume could result in more contact with the refrigerant and with all the cryoprotective components (e.g., egg yolk, EDTA, citrate), allowing the homogeneous and rapid cooling of the sample [6,49]. A correct cooling rate enables cells to adapt to temperature changes, reducing the injuries originated by cold shock. It is known that spermatozoa are very sensitive to a rapid reduction in temperature from 25 to 5 °C, which induces stress in the membranes related to a reorganization of phospholipids and proteins [6,18,50,51]. This alters their functional status and permeability, affecting the function of ion channels, producing reactive oxygen species (ROS), and altering the potential of the mitochondrial membrane [11,52]. Therefore, cooling the samples in straws instead of tubes could minimize damage to the sperm during freezing and would also greatly facilitate the development of a freezing protocol for epididymal sperm samples in the field, where it is more complex to use cooling chambers at 5 °C to pack the straws after the cooling process.

Besides this, in Experiment 2, different equilibration times were studied to simplify procedures. During the equilibration time, the period following the cooling stage, sperm membranes are stabilized at 5 °C to minimize cold injuries. This is possible because this period allows water to exit and facilitates the entrance of permeable cryoprotectants (e.g., glycerol), which exert their cryoprotective effect on the sperm [35,51,52,53]. In several studies of other species, an association has previously been found between an average equilibration time (2–4 h) and the preservation of the motility and integrity of sperm membranes [52,53,54,55,56]. However, there are no published studies regarding different durations of this stage and their effect on the thawing of Iberian red deer epididymal spermatozoa, with the most frequently used equilibrium time being 120 min [6,26]. In the present study, there were differences in the NAR percentage between samples with no equilibration time and those kept at 5 °C for 120 min, but not between the middle length periods (30 and 60 min) and the 120 min group. Thus, even though there were no significant differences between the middle groups, we could observe that all these parameters tended to be better when an equilibration time was applied. Moreover, the longer equilibration time showed higher values for VSL and VAP. This shows that in Iberian red deer, a long equilibration time may be essential to ensure epididymal sperm viability, regardless of its duration.

Similarly, other research studies in Gyr bulls [57] obtained better post-thawing sperm quality when implementing an equilibration period, no matter its length. This apparent cryoresistance of the epididymal spermatozoa may be due to their morphological and biophysical properties in their lipid membrane compared to ejaculated spermatozoa, as well as their smaller size [1,8,58]. The latter would imply a higher osmotic tolerance by the epididymal spermatozoa, due to a lower water volume, facilitating the flow of cryoprotectants and water during shorter equilibrium times [8]. 

Finally, the last factor we assessed in this study was the freezing methodology, to facilitate its development under field conditions. To date, no study has evaluated different freezing techniques for Iberian red deer epididymal sperm, for which the methods used are adapted from sheep and goats [59]. In these species, the conventional techniques widely used are based on nitrogen vapor freezing, in which samples are suspended in nitrogen vapor for approximately 15 min (with the cooling speed being on average 16–25 °C/min) and then rapidly immersed in liquid nitrogen at −196 °C for storage [6,60]. Studies in some wild ruminant species such as mouflon and fallow deer have shown promising results in semen thawing using this methodology compared to other ultrarapid freezing techniques [8,61]. Herein, we compared three alternative freezing methods (B, DY, and MP) using the same semen sample packaging conditions (plastic straws) as the conventional nitrogen vapor freezing in a tank. The results showed that only the metallic plate method (MP) obtained significantly worse outcomes in post-thaw semen quality (see Figure 4). This lower sperm quality in the MP procedure may be due to the high conductivity of the metal, which would determine a faster loss of cold compared to other materials. In addition, the fact that the straws were placed directly on the metal plate would have led to a high cooling rate on the side in direct contact with the plate and a much lower cooling rate on the top side, which was not in direct contact. Therefore, no homogenous freezing would occur [62].

In contrast, the polystyrene-box-frozen samples gave comparable results without significant differences to the control group (C), which could make them a suitable alternative to tank-freezing with liquid nitrogen. Some studies have reported similar results between dry ice and nitrogen vapor liquid [63]. It should be said that, despite the recent interest in ultrafast freezing techniques, also known as vitrification techniques, as they are easy to perform and low-cost, they have not been considered in the deer species. This is not only because of the significant effect that high concentrations of cryoprotectants have on the post-thaw quality of the sperm [8,62,64], but also because they are not very profitable. In most cases, the vitrification techniques involve freezing a small volume of semen, which requires more advanced reproductive techniques such as ICSI, which needs only one spermatozoon per oocyte to be fertilized. On the other hand, in most wild ruminant species, such as the Iberian red deer, artificial insemination (AI) and in vitro fertilization (IVF) are the most frequently used reproductive techniques, which require a larger volume of semen sample for their execution. For this reason, we thought that freezing in a polystyrene box would present some advantages, as it requires a material that can be handled in field conditions, which facilitates the preservation of sperm samples collected outside the laboratory for species where vitrification is not an option or needs to be optimized.

## 5. Conclusions

Typically, the collection and processing of Iberian red deer sperm samples are carried out on captured males under field conditions. In this work, we introduce variations to the standard technique to develop a specific protocol that is both easy to develop under field conditions and preserves sample quality. The best conditions, according to our findings, were: (1) storing samples in straws prior to refrigeration, as it minimizes cell damage due to temperature fluctuations; (2) considering a long equilibration time for the samples; (3) freezing samples in liquid nitrogen vapors using a polystyrene box, which has some advantages, as it is cheaper and more manageable. Thus, the protocol established can be improved and made more specific and more appropriate for application under field conditions, which would ultimately help establish a Genome Resource Bank for deer species, making possible not only the genetic improvement of the Iberian red deer but also the preservation of other related endangered subspecies.

## Figures and Tables

**Table 1:** Effects of storage methods (tubes of 15 mL or 0.25 mL straws) during refrigeration of Iberian red deer epididymal spermatozoa on kinematics parameters. Data represented as mean ± SEM. VCL—curvilinear velocity (µm/s); VSL—rectilinear velocity (µm/s); VAP—velocity for the corrected trajectory (µm/s); LIN—linearity (%); ALH—lateral head displacement (µm). Same letter within columns indicate not significant differences (*p* ≥ 0.05).

Storage Method	VCL	VSL	VAP	LIN	ALH
Tube	105.70 ± 9.06 ^a^	32.52 ± 4.97 ^a^	64.12 ± 9.94 ^a^	30.49 ± 3.51 ^a^	4.28 ± 0.45 ^a^
Straw	110.63 ± 21.00 ^a^	32.63 ± 4.97 ^a^	66.97 ± 13.91 ^a^	30.04 ± 3.55 ^a^	4.49 ± 0.83 ^a^

**Table 2:** Effects of different equilibration times (0, 30, 60, and 120 minutes) on sperm motility CASA parameters of Iberian red deer epididymal spermatozoa. Data represented as mean ± SEM. VCL—curvilinear velocity (µm/seg); VSL—rectilinear velocity (µm/seg); VAP—velocity for the corrected trajectory (µm/seg); LIN—linearity (%); ALH—lateral head displacement (µm). Different letters within columns indicate significant differences (*p* ≤ 0.05).

Equilibration Time	VCL	VSL	VAP	LIN	ALH
0 min	73.43 ± 4.18 ^a^	22.48 ± 1.21 ^a^	44.22 ± 2.82 ^a^	31.08 ± 1.14 ^a^	3.16 ± 0.16 ^a^
30 min	82.36 ± 4.18 ^a^	25.54 ± 1.21 ^a^	51.18 ± 2.82 ^a^	30.20 ± 1.14 ^a^	3.43 ± 0.16 ^a^
60 min	86.39 ± 4.27 ^a^	26.91 ± 1.23 ^a^	54.71 ± 2.88 ^a^	30.49 ± 1.16 ^a^	3.54 ± 0.16 ^a^
120 min	85.59 ± 4.18 ^a^	28.19 ± 1.21 ^b^	55.42 ± 2.82 ^b^	31.92 ± 1.14 ^a^	3.46 ± 0.16 ^a^

**Table 3:** Effects of different freezing techniques on kinematics parameters of Iberian red deer epididymal spermatozoa. C (control—liquid nitrogen vapor in a tank), B (box—polystyrene box with liquid nitrogen inside), DY (dry ice—polystyrene box with dry ice inside), and MP (metallic plate—solid metallic plate floating on the surface of liquid nitrogen). Data represented as mean ± SEM. VCL—curvilinear velocity (µm/seg); VSL—rectilinear velocity (µm/seg); VAP—velocity for the corrected trajectory (µm/seg); LIN—linearity (%); ALH—lateral head displacement (µm). Different letters within columns indicate significant differences (*p* ≤ 0.05).

Freezing Technique	VCL	VSL	VAP	LIN	ALH
C	105.70 ± 9.06 ^a^	32.52 ± 4.97 ^a^	64.12 ± 9.94 ^a^	30.49 ± 3.51 ^a^	4.28 ± 0.45 ^a^
B	103.15 ± 17.74 ^a^	32.18 ± 4.71 ^a^	64.12 ± 9.94 ^a^	30.93 ± 2.25 ^a^	4.18 ± 0.79 ^a^
DY	92.60 ± 15.42 ^a^	28.59 ± 3.82 ^a^	59.11 ± 9.44 ^a^	30.42 ± 2.48 ^a^	3.77 ± 0.60 ^a,b^
MP	54.24 ± 28.46 ^b^	15.97 ± 7.81 ^b^	31.12 ± 16.84 ^b^	28.86 ± 4.47 ^a^	2.44 ± 1.05 ^b^

## Data Availability

The results can be shared on demand.
